# 分子靶向治疗在肺鳞癌中的研究进展

**DOI:** 10.3779/j.issn.1009-3419.2013.12.10

**Published:** 2013-12-20

**Authors:** 丽 马, 树才 张

**Affiliations:** 101149 北京，首都医科大学附属北京胸科医院，北京市结核病胸部肿瘤研究所 Department of Medical Oncology, Beijing Chest Hospital, Capital Medical University, Beijing Tuberculosis and Thoracic Tumor Research Institute, Beijing 101149, China

**Keywords:** 肺肿瘤, 鳞状细胞癌, 靶向治疗, Lung neoplasm, Squamous-cell cancer, Targeted therapy

## Abstract

肺鳞癌（squamous-cell lung cancer, SQCLC）是一种常见的肺癌病理类型，全世界每年约40余万人死于肺鳞癌，发病与吸烟密切相关。然而，研究表明，在肺腺癌中有明显疗效的靶向药物却无法让肺鳞癌患者获益，如人表皮生长因子受体（epidermal growth factor receptor, EGFR）抑制剂、间变性淋巴瘤激酶（anaplastic lymphoma kinase, ALK）抑制剂等。通过大量基因组学研究表明，纤维母细胞生长因子受体1（fibroblast growth factor receptor 1, *FGFR1*）基因扩增和盘状结构域受体2（the discoidin domain receptor 2, *DDR2*）基因突变等都可能成为新的用于治疗肺鳞癌的潜在药物分子靶点。此外，肺鳞癌患者基因组中也存在特异性的基因变异位点，这些改变在肺鳞癌细胞周期调控、氧化应激反应、细胞凋亡和鳞状上皮分化过程中发挥了重要作用，也可能为寻找候选分子靶点提供依据。本综述通过回顾近年来肺鳞癌分子靶向治疗的相关研究，分析靶向治疗在肺鳞癌中的研究进展，使肺鳞癌的个体化靶向治疗成为可能。

## Abstract (en)

Squamous cell lung cancer (SQCLC) is one of the most prevalent subtypes of lung cancer worldwide, about 400, 000 persons die from squamous-cell lung cancer around the world, and its pathogenesis is closely linked with tobacco exposure. Unfortunately, squamous-cell lung cancer patients do not benefit from major advances in the development of targeted therapeutics such as epidermal growth factor receptor (EGFR) inhibitors or anaplastic lymphoma kinase (ALK) inhibitors that show exquisite activity in lungadenocarcinomas with EGFR mutations or echinoderm microtubule associated protein like-4 (EML4)-ALK fusions, respectively. Major efforts have been launched to characterize the genomes of squamous-cell lung cancers. Among the new results emanating from these efforts are amplifications of the fibroblast growth factor receptor 1 (*FGFR1*) gene, the discoidin domain receptor 2 (*DDR2*) gene mutation as potential novel targets for the treatment of SQCLCs. Researchers find that there are many specific molecular targeted genes in the genome of squamous-cell lung cancer patients. These changes play a vital role in cell cycle regulation, oxidative stress, cell apoptosis, squamous epithelium differentiation, may be the candidate targeted moleculars in SQCLCs. Here, we provide a review on these discoveries and their implications for clinical trials in squamous-cell lungcancer assessing the value of novel therapeutics addressing these targets.

过去的十几年中，分子靶向治疗在肺腺癌的治疗中具有里程碑式的作用，至少1/3的肺腺癌患者均能从靶向药物治疗中获益，如人表皮生长因子受体（epidermal growth factor receptor, EGFR）抑制剂吉非替尼、厄洛替尼和埃克替尼以及间变性淋巴瘤激酶（anaplastic lymphoma kinase, ALK）抑制剂克唑替尼等，但肺鳞癌却仍无明确的分子靶点指导临床实践^[1]^。最新报道陆续揭示了新的肺鳞癌相关基因改变。本文旨在综述近年来肺鳞癌分子靶向治疗的相关研究，从肺鳞癌流行病学特征、肿瘤发生及生物学差异、潜在分子靶点的发现与研究进展、临床研究的进展等方面分析靶向药物应用于肺鳞癌治疗中的临床价值。

## 1 肺鳞癌流行病学和病理学特征

肺鳞癌约占非小细胞肺癌的20%-30%，与吸烟密切相关^[2]^。肺鳞癌的组织病理特征为细胞角质化、细胞间桥呈珍珠状，肿瘤细胞有大量胞质，不规则胞核，核仁较小^[3]^。临床上可通过手术切除的肿瘤标本、细针穿刺细胞学或支气管刷获取的细胞学或组织学标本用于诊断。通过免疫组织化学法分析癌基因*p63*和甲状腺转录因子-1（thyroid transcription factor-1, TTF-1）的表达水平协助诊断肺鳞癌。肺鳞癌均表达P63而不表达TTF1^[3]^。最近证据^[4]^表明P40，一个P63同型异构体抗体，对鳞状细胞核特异性更高（ΔNP63），比传统P63抗体更敏感。还有一些鳞状细胞免疫标志物也被广泛应用于临床实践，如高分子量的细胞角蛋白5/6（cytokeratin 5/6, CK5/6），但由于组织学的差异和染色分级系统不完善，其诊断的特异性和敏感性还有待进一步研究^[5]^。此外，腺鳞癌在非小细胞肺癌约占0.4%-4%，常见于吸烟、*EGFR*野生型和无*ALK*基因变异的患者^[6]^。因此，临床医生需进一步鉴别鳞癌和腺鳞癌。由于分子检测和临床诊断多基于病理组织学的鉴定，组织学的误差可能会导致一些潜在的病例错失接受靶向治疗的机会，因此临床医生也应总结临床特征，加强与病理科医师的协作，保证诊断的准确性。

## 2 肿瘤发生和生物学差异

### 2.1 肿瘤发生的基因变异

临床治疗疗效的差异提示肺鳞癌的生物学特征与肺腺癌存在差异。新的分子检测方法的出现使我们探索到鳞癌基因序列的特征。长期暴露于烟草导致上呼吸道正常上皮形态发生改变。支气管上皮、粘膜的变化使基底细胞增生，鳞状上皮化生，导致肿瘤的发生。最早的改变发生在正常上皮包括染色体3p区域性等位缺失（3p21, 3p22–24, 3p25），染色体9p21[细胞周期依赖性激酶抑制基因（cyclin-dependent kinase inhibit gene, CDKN2A）]和8p21-23缺失^[7]^。这些变异均伴随着17p13[抑癌基因（P53 tumor protein, TP53）]、13q14[视网膜母细胞瘤基因1（retinoblastoma 1, RB1）]的缺失。证据^[8]^表明，除了上述位点变异，肺鳞癌可能还存在一些信号通路的改变。如磷脂酰肌醇3-激酶（phosphatidylinositol-3-kinase, PI3K）通路，泛素连接酶复合体衔接蛋白1（Kelch-like ECH-associated protein1, KEAP1）/小鼠核因子E2相关蛋白2（Rat nuclear factor erythroid 2-related factor 2, NFE2L2）通路等。研究者正试图从中探索特异性鳞癌分子靶点，为开发新的药物提供依据。

#### 2.1.1 Y染色体性别决定区相关高速泳动族框因子（sry-related HMG box-containing, *SOX2*）基因扩增

*SOX2*基因是一个最新发现的与肺鳞癌患者生存密切相关的癌基因，可以控制胚胎干细胞多能转化和气管支气管上皮的形成。同时，SOX2参与了从正常上皮到恶性肿瘤的发展，促进鳞癌组织标志物的表达，如P63^[9]^。通过比较基因组杂交技术（comparative genomic hybridization, CGH）发现，60%-80%的鳞癌患者存在*SOX2*基因扩增，Wilbertz等^[10]^通过两个独立队列研究发现了SOX2扩增在非小细胞肺癌中的作用。免疫组织化学染色分析指出，与肺腺癌相比，SOX2的表达在肺鳞癌中明显升高（*P* < 0.001），荧光原位杂交技术检测提示大多数肺鳞癌肿瘤组织中SOX2低水平扩增（68%，143/210例），而在肺腺癌中只发现6%例（13/208例）有SOX2低水平扩增。此外，高水平扩增只出现在肺鳞癌中（8%，16/210例）。SOX2蛋白高表达患者总生存期（overall survival, OS）相对延长（*P*=0.036），但SOX2高水平扩增与OS延长无明显相关（*P*=0.078）。研究^[8, 11]^表明，仅*SOX2*基因扩增虽不是组织恶性转化的必要条件，但可能参与了肿瘤的发生，并促进鳞癌细胞的增殖、分化、侵袭和迁移。目前虽然未明确SOX2扩增是一个特异性的分子靶点，但由于这些肿瘤的细胞周期蛋白D1（cyclinD1）水平均过表达，因此，细胞周期抑制剂可能是一个潜在可行的治疗手段^[12]^。

#### 2.1.2 TP63扩增

*P63*是具有转录活性的P53的靶基因，而P63截断体被认为是鳞癌细胞必要因子，是促进鳞癌发生发展的癌基因。截断型ΔNP63α片段变异体普遍表达于鳞状上皮和TP63扩增的肺癌细胞中。肺鳞癌中TP63既有基因扩增也有蛋白过表达^[13]^。一项Massion的研究^[13]^通过荧光原位杂交技术检测出88%（191/271例）肺鳞癌有TP63扩增。在鳞状细胞恶性转化前就可以检测到此基因拷贝数的增加，提示其在鳞癌早诊中具有潜在的病理学价值。*TP63*基因扩增和过表达与肺鳞癌患者生存时间密切相关^[14]^。

#### 2.1.3 *KEAP1*/*NFE2L2*基因突变

细胞通过KEAP1-NFE2L2通路调节对氧化应激及异源化合物应激的反应。研究表明，*KEAP1*基因突变在非小细胞肺癌中约占19%，其中大部分是肺腺癌^[15]^。NFE2L2是一个转录活性因子，参与谷胱甘肽合成，活性氧自由基灭活，抑制异源化合物活性。NFE2L2高表达和KEAP1低表达与非小细胞肺癌生存时间短密切相关^[16]^。研究^[16]^表明，*NFE2L2*突变主要发生在肺鳞癌中，与吸烟有关。在两个KEAP1相连的区域出现*NFE2L2*突变，激活NFE2L2转录活性，促进细胞增殖分化^[17]^。Shibata^[18]^分析了NFE2L2点突变出现在10.7%（11/103例）原发非小细胞肺癌（主要是肺鳞癌）。一项研究^[19]^报道了mTOR激活存在于*NFE2L2*突变的肿瘤细胞系中，mTOR抑制剂西罗莫司（sirolimus）可在体内体外抑制该细胞的增殖。但目前尚无特异性的NFE2L2抑制剂。此外，其他基因可能在KEAP1-NFE2L2通路中也起一定修饰作用。Sarkaria^[20]^发现鳞状细胞相关蛋白（defective in cullin neddylation 1, domain containing 1, DCUN1D1）扩增出现在48%（21/44例）原发肺癌中，其中63%（20/32例）肺鳞癌患者中存在DCUN1D1的过表达，而在腺癌中只发现了8%（1/12例），两者具有统计学差异（*P*=0.004），提示其在鳞癌中的相对特异性表达，但仍需进一步明确其在鳞癌肿瘤发生发展中的作用。

### 2.2 潜在驱动基因

#### 2.2.1 磷脂酰肌醇3-激酶/蛋白激酶B/哺乳动物雷帕霉素靶蛋白（phosphatidylinositol-3-kinase/protein kinase B/mammalian target of Rapamycin, PI3K/Akt/mTOR）信号通路

PI3K/AKT/mTOR信号通路是一个调节细胞分化、代谢、增殖、血管发生的重要传导通路。研究^[20]^表明PI3K/PTEN/AKT/mTOR通路的异常在肺鳞癌中比肺腺癌更常见。*PIK3CA*突变率在鳞癌中约占3.6%-6.5%，突变发生在第9和20外显子^[21]^。Okudela等^[22]^通过荧光原位杂交技术检测到43%（12/28例）日本肺鳞癌患者存在PIK3CA基因扩增，而Ji等^[23]^通过PCR技术检测到42%（40/95例）中国肺鳞癌患者存在*PIK3CA*基因扩增。此外，AKT1是PI3K/AKT信号途径的活性中心，*AKT1*的E17K突变导致PI3K/AKT信号通路的活化，具有抗肿瘤细胞凋亡，促进肿瘤细胞的增殖和分化的作用^[24]^。报道称*AKT1*突变存在肺鳞癌中约7%（5/73例），但在肺腺癌中尚未发现，提示在肺鳞癌中AKT1 E17K突变可能作为治疗肺鳞癌特异候选靶向基因发挥更重要的作用。*PTEN*是肿瘤抑制基因，可负调节PI3K-AKT-mTOR通路，PTEN缺失可以激活此通路，促进细胞生长和增殖。有研究^[25, 26]^报道了*PTEN*表达缺失与*PTEN*甲基化分别发生在24%（30/125例）和35%（7/20例）的非小细胞肺癌中，指出*PTEN*基因突变在肺鳞癌中占10.2%（6/59例），明显高于肺腺癌（1.7%，2/117例）（*P*=0.02）。来自纪念斯隆-凯特琳癌症中心（MSKCC）的研究^[27]^发现，经免疫组化确诊的肺鳞癌中未发现*EGFR*/*KRAS*突变，而发现4%（4/95例）*PIK3CA*突变和1%（1/95例）*AKT1*突变，为*PIK3CA*突变和*AKT1*突变作为肺鳞癌的靶向基因提供证据。此外，mTOR高表达于非小细胞肺癌中，其中肺鳞癌与肺腺癌的表达无统计学差异（*P*=0.65），与肿瘤侵袭性高、预后差明显相关^[28]^。mTOR抑制剂（依维莫司）体内体外研究均可抑制肿瘤的生长，其应用于肺癌的Ⅰ期/Ⅱ期临床研究获得良好的安全性和有效性^[29]^。

一些PI3K通路的抑制剂正在用于实体瘤治疗研究中，包括PI3K不同异构体的抑制剂，AKT1和mTOR抑制剂，PI3K/mTOR双靶点抑制剂。这些研究的要点集中在肺腺癌中*PIK3CA*基因突变与其他癌基因的变异同时存在的情况下，如*KRAS*、*BRAF*、*EGFR*和*EML4-ALK*的基因变异。部分靶点陆续应用于肺鳞癌中，研究者试图通过多靶点联合抑制达到更好疗效已经成为目前靶向治疗的新方向^[21]^。

#### 2.2.2 纤维母细胞生长因子受体1（fibroblast growth factor receptor 1, FGFR1）扩增

FGFR是一个酪氨酸激酶跨膜受体，参与胚胎的发育、细胞增殖、分化和血管生成。FGFR家族有四个成员（FGFR1、FGFR2、FGFR3、FGFR4），通过扩增、突变或易位而导致肿瘤的发生。2010年，FGFR1扩增首次发现于肺鳞癌中，吸烟可能通过破坏FGFR1蛋白的编码基因而与肺鳞癌的发生密切相关^[30]^。近期Weiss等^[31]^在155例原发肺鳞癌标本中，通过单核苷酸多态性（single nucleotide polymorphism, SNP）技术发现22%例标本存在FGFR1扩增，而581例非鳞癌患者中，仅1%存在*FGFR1*基因扩增。结果显示，FGFR1扩增可能是肺鳞癌特有的分子标志。进一步动物试验中还指出在裸鼠致瘤模型中运用FGFR1抑制剂PD173074可使存在FGFR1基因扩增的肺鳞癌小鼠的肿瘤明显缩小。一些选择性FGFR1酪氨酸抑制剂（AZD4547、BGJ398）早期临床试验（ClinicalTrail.gov NCT01795768）亦在进行中^[8]^。

#### 2.2.3 盘状结构域受体（discoidin domain receptor tyrosine kinase 2, *DDR2*）突变

DDR是一个酪氨酸激酶受体，调节细胞粘连、增殖，通过与配体胶原连接调节胞外重建机制。非小细胞肺癌中DDR1的上调与无病生存时间和总生存时间延长密切相关，尤其是鳞癌^[32]^。2011年，Hammerman等^[33]^通过测序法检测了290例肺鳞癌肿瘤组织和细胞系明确*DDR2*突变率为3.8%。进一步体外研究表明针对DDR1和DDR2的多激酶抑制剂达沙替尼可以抑制*DDR2*突变的肿瘤细胞增殖和分化。早期研究还发现了1例EGFR野生型肺鳞癌患者接受达沙替尼和厄洛替尼治疗后影像学上病灶明显缩小。而将治疗前肿瘤组织进行DNA测序发现*DDR2*激酶区突变（S768R）^[34]^。肺鳞癌*DDR2*突变率虽然不高，接近于肺腺癌*EML4-ALK*融合基因的发生率，但若进一步研究发现肺鳞癌*DDR2*突变者对达沙替尼确实有效，将会对该亚型患者带来革命性福音。

#### 2.2.4 *EGFR* Ⅷ变异

研究^[35]^表明，虽然*EGFR*敏感突变（19外显子缺失和L858R点突变）在鳞癌中少见，但可以在部分患者中找到另外一种*EGFR*突变类型：外显子2-7的第Ⅲ类*EGFR*缺失突变（*EGFR* Ⅷ）。这个区域缺失导致配体二聚体形成和促进磷酸化激活。这种突变在肺鳞癌的突变率约5%-8%。小样本研究^[36]^表明*EGFR* Ⅷ突变并不是一个对可逆型TKI敏感的突变类型。临床研究和实践中，大部分*EGFR*突变且EGFR-TKI受益者是肺腺癌患者，此突变的鳞癌患者能否获得与腺癌同样的疗效尚缺乏大样本研究。未来的研究重点应探索不可逆EGFR/Her2抑制剂Neratinib、Afatinib在肺鳞癌中的作用以及*EGFR* Ⅷ突变能否作为此类药物疗效预测的分子标志。

## 3 总结

第二代基因测序技术的快速发展使了解不同种类疾病的基因组学特征成为可能，使肺鳞癌的个体化治疗紧随肺腺癌的发展，成为目前关注的热点之一。目前可行的靶向治疗在*PIK3CA*突变（20%-30%）、FGFR1扩增（20%）和*DDR2*突变（4%）已经成为个体化治疗靶点的选择^[8]^。*DDR2*突变和FGFR1扩增导致相应跨膜受体信号下调增加。*PIK3CA*、*PTEN*和*AKT*基因变异导致PI3K通路激活。*KEAP1*或*NFE2L2*突变导致NFE2L2介导的细胞保护基因表达增加。SOX2扩增导致控制肿瘤生长的SOX2介导基因激活。此外，突变基因还包括*TP53*、*CDKN2A*、*MLL2*、*NOTCH1*、*RB1*和*HLA-A*。最新研究^[37]^表明免疫治疗在肿瘤治疗中也发挥了重要的作用，如程序性死亡分子1（programmed death 1, PD1）和细胞毒T淋巴细胞相关抗原4（cytotoxic T lymphocyte-associated antigen-4, CTLA4）抑制剂。重要的通路包括NFE2L2/KEAP1、PI3K/AKT、CDKN2A/RB1，这些基因也参与鳞状细胞的恶性转化。针对这些新靶点的临床研究正在开展，虽然存在很多争议和疑惑，但这些发现和探索将会为肺鳞癌的个体化靶向治疗提供新的思路和诊疗方案。
